# Modification of Epigenetic Histone Acetylation in Hepatocellular Carcinoma

**DOI:** 10.3390/cancers10010008

**Published:** 2018-01-03

**Authors:** Kwei-Yan Liu, Li-Ting Wang, Shih-Hsien Hsu

**Affiliations:** 1 Graduate Institute of Medicine, College of Medicine, Kaohsiung Medical University, Kaohsiung 80708, Taiwan; a52t5213b@hotmail.com (K.-Y.L.); innywang91104@gmail.com (L.-T.W.); 2 Department of Medical Research, Kaohsiung Medical University Hospital, Kaohsiung 80708, Taiwan

**Keywords:** AHR, HDAC8, hepatocellular carcinoma

## Abstract

Cells respond to various environmental factors such as nutrients, food intake, and drugs or toxins by undergoing dynamic epigenetic changes. An imbalance in dynamic epigenetic changes is one of the major causes of disease, oncogenic activities, and immunosuppressive effects. The aryl hydrocarbon receptor (AHR) is a unique cellular chemical sensor present in most organs, and its dysregulation has been demonstrated in multiple stages of tumor progression in humans and experimental models; however, the effects of the pathogenic mechanisms of AHR on epigenetic regulation remain unclear. Apart from proto-oncogene activation, epigenetic repressions of tumor suppressor genes are involved in tumor initiation, procession, and metastasis. Reverse epigenetic repression of the tumor suppressor genes by epigenetic enzyme activity inhibition and epigenetic enzyme level manipulation is a potential path for tumor therapy. Current evidence and our recent work on deacetylation of histones on tumor-suppressive genes suggest that histone deacetylase (HDAC) is involved in tumor formation and progression, and treating hepatocellular carcinoma with HDAC inhibitors can, at least partially, repress tumor proliferation and transformation by recusing the expression of tumor-suppressive genes such as *TP53* and *RB1*.

## 1. Introduction

Hepatocellular carcinoma (HCC), one of the most common malignancies of primary liver cancer, results in 500,000 global deaths every year. Because of the challenges associated with early discovery, most patients with HCC are diagnosed in advanced stages of the disease [1]. According to the Barcelona Clinic Liver Cancer classification system, patients with early-stage HCC (Stage A) are optimal candidates for a radical approach including surgical resection and orthotopic liver transplantation (OLT). Only a few patients with HCC achieve acceptable outcomes with the standard curative treatment, including OLT, because of organ shortage [2]. Local ablative therapies including radiofrequency ablation, microwave ablation, laser-induced interstitial thermotherapy, and percutaneous alcohol injection (PEI) [3] are alternative treatments for those patients who cannot be treated by surgical resection or liver transplantation. Patients with HCC who have large or multinodular lesions only in the liver and have sufficient liver function (Stage B) are subjected to palliative treatment using transarterial chemoembolization. The overall survival of patients with advanced HCC (Stage C) with vascular invasion or extrahepatic spread and physical impairment, was improved by systemic therapy with sorafenib, an oral multikinase inhibitor [4]. Patients with end-stage HCC (Stage D) with severe liver damage and impaired physical status receive palliative treatment. Despite curative resection and improved treatments, many patients with HCC experience recurrence after surgery. Poor survival of patients with HCC can be largely attributed to rapid intrahepatic recurrence (IHR) due to cancer cell metastases.

HCC develops as a consequence of chronic inflammation and cirrhosis caused by viral hepatitis B and C, alcoholism, or exposure to aflatoxins [5,6]. Those risk factors lead to malignant transformation by genetic alteration. However, recent discoveries demonstrate that genetic, genomic, and epigenetic alterations should be considered as factors in HCC development [7].

## 2. Aryl Hydrocarbon Receptor

### 2.1. Function

The liver is a major organ for the metabolism of xenobiotics that stem from environmental pollution and dietary intake. Many xenobiotics and their metabolites have detrimental and tumorigenic effects on hepatocytes. The aryl hydrocarbon receptor (AHR) is a cytosolic basic helix-loop-helix/Per-Arnt-Sim chemical sensor [8]. AHR can sense various environmental stimuli such as polycyclic aromatic hydrocarbons and a diverse array of endogenous metabolites [9]. When AHR encounters ligands, it translocates from the cytoplasm to the nucleus to heterodimerize with the AHR nuclear translocator (ARNT) and activates the expression of a battery of genes containing specific xenobiotic-responsive elements. Those genes, which encode Phase I and II enzymes, are associated with the cellular detoxification process. Some of the toxic and carcinogenic metabolites that are generated in the detoxification process may play important roles in multiple stages of tumor progression.

### 2.2. AHR Regulation in HCC

The AHR influences initiation, promotion, progression, and metastasis of tumorigenesis [10]. The environmental pollutant 2,3,7,8-tetrachlorodibenzo-p-dioxin (TCDD) is an HCC carcinogen that can activate AHR as a high-affinity ligand to significantly promote AHR transcriptional activity. Mice treated with TCDD expressed hepatic fibrosis markers such as collagen type 1alpha 1 (COL1A1) and α-smooth muscle actin and promoted the development of HCC. In contrast, the AhR-mutant mice [11,12] did not develop such activity, the involvement of AhR in TCDD-induced HCC. In breast cancer, AHR activation by TCDD promotes interleukin 6 (IL-6) induction in the presence of an inflammatory signal by directly binding with the IL-6 promoter [13]. It is possible that AHR integrates in IL-6 signaling in the malignant progression of HCC [14].

Our previous studies found that AHR promotes tumorigenesis by cooperating with a newly identified proto-oncogene, intestine-specific homeobox (ISX) in HCC [15,16]. Ablation of AHR or ISX in hepatoma cells suppresses cell growth, whereas its overexpression promotes cell proliferation and leads to enhanced in vitro and in vivo tumorigenic activity. AHR targets ISX, increasing the expression of this gene and its downstream targets, which include CCND1 and E2F1. In HCC development, AHR plays a critical role in tumor cell immunosuppression. Tumor cells express programmed cell-death 1 ligand (PD-L1) and the cluster of differentiation 86 (CD86) protein binds to immune-checkpoint receptors programmed cell-death protein 1 (PD-1) and cytotoxic T-lymphocyte-associated protein 4 (CTLA-4) of CD8+ T-cell to inhibit host immune responses [17]. Our previous studies showed that liver tumors result in immune suppression by expressing PD-L1 and CD86 through induction by the AHR-ISX cascade [16]. The depletion of AHR or ISX in liver tumors converts the immune activity of CD8+ T-cells and promotes the death of liver tumor cells. Despite AHR regulation of immune-checkpoint molecules, AHR plays dual roles in tumor and immune cells. In the tumor microenvironment (TME), kynurenine (KYN) accumulation, an endogenous metabolites of tryptophan catabolism, acts as a ligand and activates AHR, which translocates to the nucleus and results in the transcription of target genes [18]. AHR activation increases the survival of tumor cells, whereas AHR activation causes tolerance in immune cells. In liver tumors, we identified the ISX-KYN-AHR axis as a self-perpetuating loop in tumorigenesis that exerts its immune-suppressive effects. ISX expression is driven by the KYN-AHR pathway, which enhances KYN accumulation in TME by direct transcriptional regulation of tryptophan catabolic enzymes, indoleamine 2,3-dioxygenases 1 (IDO1), and tryptophan 2,3-dioxygenase (TDO2). KYN-AHR-ISX axis could amplify this feedback loop to build up immune-checkpoint- and AHR-dependent immune-suppressive effects in HCC tumorigenesis.

Despite the KYN-AHR-ISX axis, we also found that AHR can promote tumorigenesis by targeting histone deacetylase (HDAC) expression and by altering epigenetic regulation [19].

## 3. AHR Regulates Epigenetic Histone Acetylation in HCC

In eukaryotic cells, genomic DNA is packaged into chromatin by the four core histones (H2A, H2B, H3, and H4) and the linker histone (H1) [20]. After posttranslational histone modification was reported by Allfrey et al. in the early 1960s [21], a number of studies have shown that histone acetylation and deacetylation are dynamic processes that activate or inhibit gene expression, and those regulations require histone acetyltransferase (HAT) or HDAC [22]. Dysregulation in the balance of HAT and HDAC is associated with developmental defects, disease, and cancer [23].

**Figure 1:**
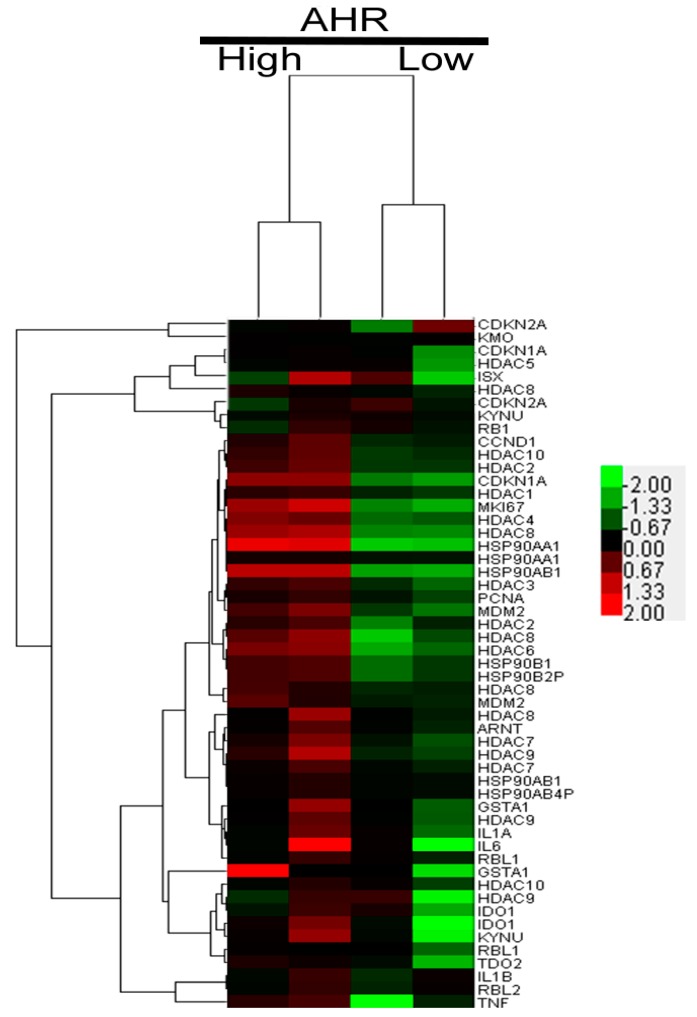
Heat map analysis of cDNA microarray data of “high” *AHR* HCC tumors compared with data from the paired healthy liver tissue. Multiple high expressions of HDAC mRNA were detected in HCC tumors with high AHR expression.

In our previous work, we examined the mRNA expression level by cDNA microarray in HCC and peripheral normal tissue of patients (Figure 1). We clustered high and low AHR expression and found high expression levels of HDAC in HCC patients with high expression of AHR, including HDAC1, HDAC2, HDAC3, HDAC4, HDAC7, HDAC8, HDAC9, and HDAC10. This suggested a correlation between AHR and HDAC overexpression and HCC development. Tumor progression was associated with an imbalance in the acetylation and deacetylation of histones and increasing the HDAC levels potentially repressed the tumor suppression genes RB1 and CDKN2A, initiating tumor formation. It has been known that HDAC8 is a direct target of AHR [19], but the regulation of AHR and HDAC in HCC needs to be identified. Recent research has demonstrated the function of HDACs within different tumor formation periods.

### 3.1. HDAC Classification

In humans, HDACs are classified into four classes based on their sequence similarities. Class I, II, and IV HDACs are numbered according to their order of discovery. Class I HDACs, including HDAC1, HDAC2, HDAC3, and HDAC8, are similar to the yeast Rpd3protein. These Class I HDACs have highly conserved deacetylase domains and extensive amino acid sequences to identify with each other. In cells, Class I HDACs can be localized within the nucleus, the cytoplasm, or some cellular organelles. It is suggested that the function and regulatory activities of Class I HDACs are not yet fully identified.

Based on the sequence of yeast Hda1 protein, HDAC4, HDAC5, HDAC6, HDAC7, HDAC9, and HDAC10 have been identified as Class II HDAC proteins. With the exception of catalytic domains that are conserved with Class I HDACs, the Class II HDAC proteins contain additional sequence domains. By comparing the homology of Class II HDAC proteins, both HDAC6 and HDAC10 contain a putative second catalytic domain not found in other HDACs and are, therefore, distinguished as Class IIb. Some of the Class II HDACs are localized to the cytoplasm and serve extranuclear functions.

The Class III proteins (SIRT1, SIRT2, SIRT3, SIRT4, SIRT5, SIRT6, and SIRT7) and the yeast Sir2 protein share similar sequences. These sirtuins contain about 22–50% identical amino acid sequence and 27–88% identical conserved catalytic domains. In contrast to other HDACs, sirtuins have mono-ADP-ribosyltransferase for enzymatic activity. An interesting feature of sirtuins is their cellular localization. SIRT1 and SIRT2 are in the nucleus and cytoplasm, SIRT3 is in the nucleus and mitochondria, SIRT4 and SIRT5 are only in the mitochondria, SIRT6 is only in the nucleus, and SIRT7 is in the nucleolus. According to their localization, sirtuins could have nonhistone substrates, at least in eukaryotes.

HDAC11, a Class IV protein, shares catalytic domains that are similar to both Class I and II proteins. HDAC11 is required for the protein stability of DNA replication factor CDT1 and the expression of interleukin 10 [24,25]. However, HDAC11 function is not extensively understood in comparison with other HDACs.

### 3.2. HDAC in HCC

In mammals, 18 known HDAC homologs were categorized into Classes I, IIa, IIb, III, and IV. HDAC1 and HDAC2 belong to the Class I group and are 83% identical based on amino acid sequences. This observation raises the possibility that HDAC1 and HDAC2 have redundant deacetylation for specific gene activation. For example, fructose-1,6-bisphosphatase (FBP1), a rate-limiting enzyme in gluconeogenesis, is downregulated in the presence of high levels of HDAC1 and HDAC2 expression in patients with HCC. Inhibition of HDAC1 and/or HDAC2 restores H3 lysine 27 acetylation (H3K27ac) of the FBP1 enhancer, rescuing FBP1 expression [26]. Increasing levels of FBP1 by HDAC inhibitors or knockdown suppress glucose metabolism, inhibit HCC cell growth, and reduce tumor growth. HDAC1 and HDAC2 cooperate to regulate deacetylation and facilitate metabolic changes in HCC.

Recent studies have shown that HDAC1 and HDAC2 have different functions during HCC progression. HDAC1 and HDAC2 were expressed in 156 Southeast Asian patients with HCC, and the expression of both is associated with mortality from cancer. Compared with well-differentiated tumors, HDAC1 expression (but not HDAC2 expression) is correlated with moderately and poorly differentiated tumors. Another study showed that high HDAC2 expression was correlated with poor survival in early-stage HCC as an independent predictor [27]. Diminished expression of HDAC1 and HDAC2 by small interfering RNA produced synergistically increased cell death and deceased cell proliferation in liver cancer cell lines [28]. Based on these findings in patients with HCC, HDAC1 and HDAC2 are believed to have independent roles in tumor progression. To understand these roles, we must identify HDAC1 and HDAC2 target genes by chromatin immunoprecipitation sequencing and transcriptome. For example, results obtained in a transcriptomic analysis from an HDAC2 knockdown experiment showed that HDAC2 controls gene expression that is involved in cell cycles, apoptosis, and lipid metabolism [29].

HDAC3 belongs to the Class I group and plays an important role in HCC formation. HDAC3 is expressed in liver cancer stem cells and is required for the self-renewal of liver cancer stem cells. Loss of HDAC3 expression in liver cancer stem cells decreases the expression of stem cell markers, including Nanog, OCT4, and SOX2. By examining the diethylnitrosamine (DEN)-induced hepatocarcinogenesis model, expression of HDAC3 was discovered during the initial stage and appeared consistently increased in liver tumors. It is possible that consistently expressed HDAC3 might be correlated with hepatocarcinogenesis [30].

HDAC and HCC are strongly correlated, but it is still unclear how HDACs are regulated in HCC. In our recent studies, we provided an example of the regulation of HDAC in HCC. HDAC8, a Class I HDAC, shows high expression in our cDNA microarray data and within the IHC data of patients with HCC [19]. Examinations of the associations between HCC progression and HDAC8 expression revealed a positive correlation of HDAC8 expression with HCC tumor size, invasion, and poor staging. Unexpectedly, HDAC8 expression was correlated with AHR expression, a chemical sensor with transcriptional activity. AHR or HDAC8 overexpression in liver cancer cells speeds cell proliferation. Regarding the relationship between HDAC8 and AHR, we found that AHR may promote HDAC8 expression by directly binding to the HDAC promoter. When liver cancer cells or mouse liver cells are treated with TCDD, a ligand of AHR, nuclear translocation of AHR turns on downstream genes, including HDAC8. Increasing HDAC8 expression selectively binds to the tumor suppressor RB1 promoter and represses the expression of RB1. Therefore, external cues, such as chemicals and pollutants, can alter the epigenetic dynamics, leading to hepatocarcinogenesis by stimulating the AHR-HDAC8 axis. Furthermore, HDAC8 is upregulated by SREBP-1 and results in dietary obesity models of nonalcoholic steatohepatitis(NASH) and HCC [31].

In addition to HDAC1-3 and HDAC8 from our microarray data, other HDACs have functions in HCC. HDAC5, a Class IIa HDAC, represses cell apoptosis and cell cycle arrest by altering the expression of pro- and antiapoptotic signals and critical cell cycle regulators [32]. HDAC9, a Class II HDAC, represses miR-376a, which downregulates HCC by removing the h3K18 acetylation [33].

## 4. HDAC Inhibitors as Clinical Trail of HCC

Histone deacetylase inhibitors (HDACis) could be used in cancer therapy by altering HDAC expression and/or function, potentially disrupting acetylation homeostasis in a variety of cancers. It might reverse the mistake of gene expression by reactivating the expression of tumor suppressors for cell cycle arrest, apoptosis, differentiation, and inhibition of angiogenesis and metastasis. HDACi numbers are increased and serve as a promising compound for patients with HCC (Table 1).

### 4.1. HDACi

To date, several HDACis have been developed from natural or synthetic molecules that target classical HDACs (Class I, II, and IV enzymes). HDACis interfere with the binding of HDAC to the Zn^2+^ ion, which is required for classical HDAC deacetylase activity, thereby inhibiting the enzymatic function of HDAC. HDACis can be arranged into four major groups on the basis of their molecular structures: hydroxamates, benzamides, short-chain fatty acids, and cyclic peptides. They can be further subdivided into nonselective, selective, and multipharmacological based on their specificity.

DNA methylation, which occurs mainly on cytosines located in CpG dinucleotide sequences, also functions as a dynamic epigenetic modification in vertebrate genome [43]. Aberrant DNA methylation status is associated with the development of HCC [44]. Previous studies have demonstrated that the dual mode of HDACis changes depending on DNA methylation status including rapid inhibition of enzyme activity due to obstruction by posttranslational acetylation and delayed effect on transcriptional control of DNA methyltransferase (DNMT) genes through HDACs or miRNA mechanisms [45]. Furthermore, HDACis triggered E-cadherin activation by both histone acetylation and DNA demethylation [46,47]. The antiepileptic drug valproic acid, which has also been shown to act as an HDACi, triggered active DNA demethylation and the activation of genes silenced by DNA demethylation [48]. This DNA demethylation might be caused by either the inhibition of maintenance DNA methyltransferase during replication, caused by the triggering of site-specific repair activity or by active site-specific or general demethylation. However, how HDACis alter the demethylation mechanism in HCC is not fully understood and needs further clarification.

### 4.2. Clinical Therapy of HDACi in HCC

Clinical and basic studies suggest that there are several classes of HDACis that have potential anticancer activities, but only in cutaneous T-cell lymphoma (CTCL) and peripheral T-cell lymphoma are HDACis applied in clinical therapy. Until now, four HDACis, which have been approved by the US Food and Drug Administration (FDA), are also tried for the treatment of HCC. Other unapproved uses of HDACi are listed in Table 1. In 2006, vorinostat (SAHA, Zolinza), a hydroxamate class agent, was approved for the treatment of patients with CTCL. Vorinostat, a product containing hydroxamic acid, is a Class I/IIb/IV inhibitor that has a structure similar to trichostatin A (TSA) [49,50]. Currently, vorinostat is being examined in multiple clinical trials focusing on renal cancer, colorectal cancer, non-small cell lung carcinoma, neuroblastoma, melanoma, and lymphoma. Vorinostat treatment of HCC cell lines can activate caspase-3 to promote cell apoptosis by TRAIL-DISC activation and autophagy leading to cell death [51,52]. In addition to its automatous effects, the antitumor activity of vorinostat can alter immune cell cytotoxicity to HCC [53]. The clinicaltrials.gov website shows a Phase I trial that is examining the side effects and dosage of vorinostat when given together with sorafenib tosylate, a kinase inhibitor drug approved for patients with advanced liver cancer.

Romidepsin (depsipeptide, ISTODAX), the second HDAC inhibitor for HDAC1 and HDAC2 that is approved for the treatment of CTCL, inhibits the growth of HCC cells by inducing cell cycle arrest and apoptosis in vivo via ERK/MAPK signaling and c-Jun N-terminal kinase (JNK)/c-Jun signaling pathway [54,55,56]. There are no romidepsin clinical trials in patients with HCC currently included on the clinicaltrials.gov website.

Belinostat (PXD101), a nonselective HDAC inhibitor containing a zinc-chelating hydroxamic acid moiety, is the third approved treatment agent for CTCL [57]. In HCC cell lines and xenografts, belinostat induces apoptosis and tumor regression but does not affect normal hepatocytes as do other HDAC inhibitors [58]. Belinostat is undergoing Phase I and Phase II clinical trials in patients with advanced liver cancer [59].

Panobinostat (LBH589), the newest HDAC inhibitor, was approved for the treatment of patients with multiple myeloma [60]. In HCC cells treated with panobinostat, endoplasmic reticulum stress-mediated apoptosis and autophagy-related cell death in vitro and in vivo were induced [61,62,63]. Panobinostat alters differentiation from an undifferentiated malignant cell to a more differentiated benign cell [64]. Panobinostat suppressed the expression of oncogenic miRNAs and promoted the maturation of the tumor suppressor miRNA in hepatocellular carcinoma cells [65,66]. Panobinostat can inhibit HCC proliferation and metastases by repressing the gankyrin/STAT3/Akt pathway [67] and can block the angiogenic properties of HCC by altering the CTGF signaling pathway [68]. Combination treatment involving sorafenib tosylate and panobinostat decreased vessel density, decreased tumor volume, and increased survival in HCC xenografts [69]. The efficacy of panobinostat should be tested in future clinical trials.

### 4.3. Combination Therapy Involving HDACi

To improve the efficacy of therapies for HCC, several studies have investigated and reported the synergistic effects of treatment with HDACis and various chemotherapeutic drugs or antitumor agents. The anti-proliferative effects on human HCC-derived cell lines are enhanced by combination treatment with the HDACi suberoylanilide hydroxamic acid (SAHA) and the demethylating agent 5-aza-2′-deoxycytidine (5-aza-dC) compared with separate treatment with each agent [70]. Combination treatment with CDK-inhibitor CYC-202 and HDACi MS-275 has a better pro-apoptotic effect than treatment with each agent independently by shifting the Bax [B-cell lymphoma 2 (Bcl-2) associated X]/Bcl-2 ratio and breaking down the mitochondrial transmembrane potentials [37]. Phosphorylation of retinoid X receptor-alpha (RXRalpha) is correlated with the development of HCC. Acyclic retinoid (ACR), which inhibits RXRalpha phosphorylation, can block the growth of HCC cells. The HDACi valproic acid (VPA) and ACR synergistically decrease the phosphorylation of RXRalpha, resulting in the accumulation of the acetylated histone proteins H3 and H4, promotion of the arrest of the G(0)–G(1) cell cycle, induction of apoptosis, and suppression of cell growth in HepG2 cells [71]. The expression of poly (ADP-ribose) polymerases (PARPs) is significantly increased in human HCC, and the inhibition of PARP-1 decreases HCC growth. Treatment with both SAHA and the PARP inhibitor Olaparib or another PARP inhibitor (PJ34) increases apoptosis and results in accumulating unrepaired DNA damage, which depends on homologous recombination deficiency in the HCC cell lines [72,73]. Fenretinide is one of the clinically tested retinoids, and it induces apoptosis in Huh7 cells, but not in HepG2 cells. Combining fenretinide with the HDACi TSA or scriptaid can not only enhance fenretinide-induced apoptosis in Huh7 but also convert the sensitivity for fenretinide-induced apoptosis in HepG2 cells [74].

In liver cancer, sorafenib acts by inhibiting the serine-threonine kinases Raf-1 and B-Raf and the receptor tyrosine kinase activity of vascular endothelial growth factor receptors (VEGFRs) 1, 2, and 3 and platelet-derived growth factor receptor β (PDGFR-β). Its use in the treatment of HCC was approved by the FDA in 2007. The current study suggests that the inhibitory activity of sorafenib also targets histone deacetylases in hepatocellular carcinoma cells [75]. A low-dose combination of SAHA with sorafenib induces apoptosis by the diminished expression of multiple antiapoptotic proteins in HCC [76]. In addition, sorafenib represses activation of the NF-κB pathway, which is induced by SAHA treatment, resulting in the synergistic antitumor activity of combination treatment in HCC [77]. The combined use of panobinostat and sorafenib in treatment has also been found to reduce tumor volume and prolong survival in HCC xenografts [69], and this combination treatment was also described in two case reports of patients with HCC [78,79].

Inhibition of the proteasome causes decreased proliferation and migration, and the induction of apoptosis. SAHA in combination with bortezomib, a proteasome 26S inhibitor, leads to enhanced apoptosis in hepatoma cells [80], and the combination of belinostat and bortezomib inhibits cell proliferation and induces apoptosis [81].

Finally, the combination of radiotherapy with HDACi treatment synergistically induces apoptosis. AR-42, a phenylbutyrate-derived HDACi, enhances radiation-induced cell death by blocking the DNA end-binding activity of Ku70, which is involved in DNA repair, telomere maintenance, and apoptosis [82]. In orthotopic and ectopic xenograft models, this combination treatment reduced tumor growth. In addition, combining VPA with the effect of photon radiotherapy prolonged proton-induced DNA damage, augmented proton-induced apoptosis, increased the proton-induced production of intracellular reactive oxygen species, and suppressed the expression of nuclear factor erythroid-2-related factor 2 (NRF2), which is a key transcription factor regulating the antioxidant response [83].

## 5. Discussion

In patients with HCC, epigenetic imbalance can be the start of tumorigenesis. HDACs play multiple roles in initial tumor formation and progression. Each HDAC has a specific and redundant function for regulating different downstream gene expressions. The reason for HDAC upregulation in patients with HCC has been unclear until now. In our study, we found that AHR, a key regulator, promotes high HDAC8 expression. AHR is also correlated with poor prognosis. Long-term exposure to environmental pollution and its metabolites can activate AHR in the liver. This activation causes the expression of HDAC8, resulting in an imbalance of epigenetic changes and tumorigenesis. The mechanism of how HDAC targets tumor suppressor genes to promote tumorigenesis is still an interesting question with several possible explanations. Proteins that interact with HDAC, such as the transcriptional regulator, provide specific binding to regulatory regions of tumor suppressor genes. Thus, the identification of proteins that interact with HDAC may unmask the upstream signaling of HDAC and elucidate how it is translocated to specific regulatory regions during liver cancer progression.

HCC is rarely detected in early stages, when surgical removal may be possible. Recurrence after surgery is likely, and there is an immediate need to identify a suitable treatment for patients who cannot undergo surgery. Nonselective HDACis, approved by the FDA, have the potential to fight cancer cells, although much remains unknown. The nonselective HDACis’ involved extensive signaling and altered entire transcriptional machinery, but only a part of HDACs were overexpressed in patients with HCC. Recently discovered small molecule drugs are able to specifically inhibit HDAC and, therefore, appear to exert antitumor activity. In our study, specific HDAC8 inhibitors repressed hepatoma cell proliferation and transformation activity via in vitro and in vivo upregulation of RB1. Therefore, combining inhibitors that target HDACs may effectively suppress tumor growth and reverse tumor progression.

## Figures and Tables

**Table 1:** Histone deacetylase (HDAC) inhibitors in hepatocellular carcinoma (HCC) treatment (unapproved from the US Food and Drug Administration (FDA)).

HDACis	Specificity	Experimental Design	Clinical Trial of HCC	References
Resminostat (4SC-201)	Classes I and II	Patient with Hepatocelluler carcinoma	Phase II trial	[34]
Quisinostat (JNJ-26481585)	Class I and II HDACs	HCC cell lines	Preclinical	[35]
MPT0E028	HDAC1, 2, 6	Patient with Hepatocelluler carcinoma	Under Phase I trial	clinicaltrials.gov
CUDC-101	Classes I and II HDAC, EGFR, HER2	HCC cell lines	Preclinical	[36]
Entinostat (MS-275)	HDAC1, 2, 3	HCC cell lines	Preclinical	[37]
Valproic acid (VPA)	Class I and II	HCC cell lines Mouse model	Preclinical	[38,39,40]
AR-42	Class I and IIb	HCC cell lines	Preclinical	[41]
Apicidin	HDAC1, 2, 3	HCC cell lines Mouse model	Preclinical	[42]
PCI-34051	HDAC8	HCC cell lines Mouse model	Preclinical	[19]
